# Contributing to health training in low and middle income countries – global health programmes' responsibility to be sustainable and impactful

**DOI:** 10.7189/jogh.10.010310

**Published:** 2020-06

**Authors:** Timothy W Rennie, Christian John Hunter

**Affiliations:** 1 School of Pharmacy, Faculty of Health Sciences, University of Namibia, Windhoek, Namibia; 2 School of Medicine, Faculty of Health Sciences, University of Namibia, Windhoek, Namibia

There is a continued challenge of meeting the health needs of people living in low and middle-income countries that is often undermined by inadequate or inequitable health care systems within those settings [1]. These health inequalities have driven funding in settings such as sub-Saharan Africa especially in response to specific disease challenges such as the HIV epidemic [2].

However, there continues to be a chronic and sustained shortage of human resources in health in low- and middle-income countries – particularly Africa – that further exacerbates those health challenges faced [3]. As an example of this, a recent global pharmacy workforce survey estimated the average pharmacist density to be 7.36 pharmacists per 10 000 population [4]. Of the nine African countries that responded to the survey, all fell well short of the average ranging from 0.11 (Madagascar) to 3.93 (Mauritius) pharmacists per 10 000; the average was 0.94/10 000. For some of those countries there was no evidence of an increase in pharmacist density in the previous decade [4].

Healthcare workers that are trained outside their countries of origin may not return to practice instead seeking a better life in more affluent or politically stable settings (the so called ‘brain drain’) [5]. The return of foreign-trained health care professionals can present further challenges in terms of adapting their practice to their home countries – they may have been trained to different standards of practice or in a different language to make easy transition back into their home countries difficult. They may also be treated differently because of negative perceptions of their training amongst the medical fraternity and struggle to get registered or licensed to practice.

Namibia is a large, sparsely populated country in southern Africa that exhibits some of highest income inequalities in the world and faces health challenges typical of the wider region. After decades of deliberation the University of Namibia formally commenced medical training in 2009 and, a year later, pharmacy training. This started the growth of national health education training to complement nurse training that existing prior to Namibian independence in 1990. Funding agencies have added significant value and accelerated the growth of the various training programmes offered, however, the reality is that most of what has been spent on this new health training initiative (buildings, equipment, salaries) has been funded by the Government of Namibia and demonstrates the prioritisation of this national project.

What could not be provided in-country due to scarce human resources is the expertise and skills mix required for training future health care workers with a reliance on foreign faculty to complement local capacity. International faculty include representation from Uganda, Zimbabwe, Zambia, Belgium, South Africa, Nigeria, Ghana, Kenya, Tanzania, USA, and the UK. The majority of foreign faculty have committed for a number of years, alongside their Namibian colleagues, integrating into the local institution; this includes visiting faculty who have been similarly committed, some for the life-span of the Faculty of Health Sciences. In addition, there are practice-based (part-time) faculty, Namibian and non-Namibian, who assist in the clinical training of students in public and private health facilities.

We assert that probably the most significant impact that can be seen in improving health outcomes in a sustainable way is by increasing the quantity and quality of health care workers in low- and middle-income countries through in-country education and training programmes. A sensible and pragmatic approach is to work with and support national institutions to build capacity not just in the training of health care workers but also in building capacity within those national training institutions to deliver quality medical and health education.

However, there continue to be significant challenges in ensuring the quality of training, for example, in undergraduate, postgraduate, internship and technical programmes that relates to ensuring the necessary skills-mix amongst faculty, adequacy of curricula, teaching materials and assessments, and the logistics of student placements and electives. Additional coordination and hosting of visiting students or residents from foreign programmes puts significant further strain on our academic systems without a return on the investment in time and effort [6].

Parallel to the funding focus in low- and middle-income countries (LMICs), or arguably as a direct consequence of funding focus on LMICs, has been a significant increase in what are generally termed ‘Global Health’ programmes, with training institutions usually located in high income countries. These often send students to different global settings to broaden the depth and breadth of their training and increase exposure particularly in disease challenges that present more frequently in sub-Saharan Africa, for example [7]. A review of global health training activities by medical specialties in 2013 showed that Africa topped the list of global health training sites [8]. For an individual these experiences are likely to have an impact on worldview and even medical practice; they are also likely to increase the future opportunities for research and publication leading to increased competitiveness in employment or promotion. What is less clear is any lasting positive impact in low and middle-income country settings that are the target of global health programme rotations – health care services may come to rely on a predictable circulation of global health ‘residents’ which may thwart efforts to increase local capacity and expertise. The visiting residents are also likely to add significant administrative burden on low and middle-income country institutions in terms of accommodation, safety, transport, orientation, visa issues etc., potentially at the expense of local students or residents. Our experience is also that the faculty accompanying the visiting residents may be unprepared, misguided or otherwise unsuitable for proper mentorship. While some Global Health programmes have made an attempt to address the one-sidedness of visiting residents by accommodating local trainee’s in their programmes, this appears to be the exception rather than the rule. We acknowledge the call for governance in this situation although the emphasis still appears to be more on the outreach institution perspective [9].

**Figure:**
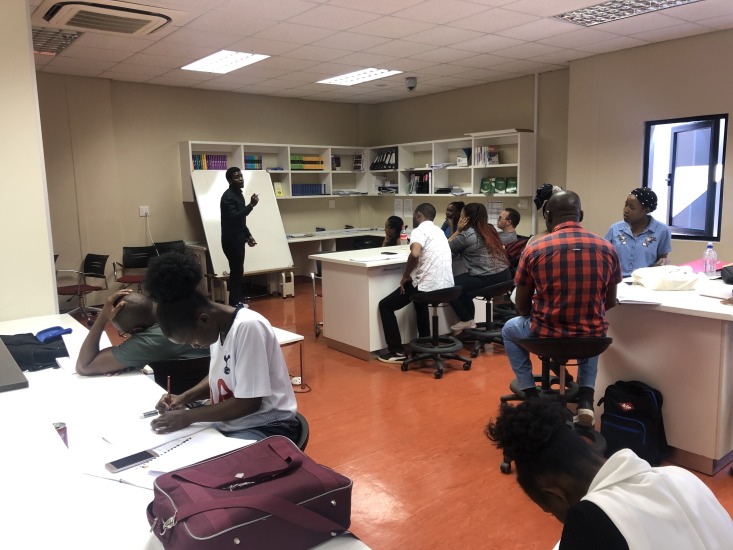
Photo: Building sustainable capacity in Namibia: Recent pharmacy graduate training Pharmaceutical Technicians in new School of Pharmacy (from the collection of Timothy Rennie, used with permission).

We challenge this approach and suggest an alternative model to propose a more integrated and equal engagement between LMIC and non-indigenous institutions. One way of improving the approach would be through a sustained commitment of academic assistance from visiting faculty that does not come with a price tag or additional significant commitments; we have welcomed a number of faculty who chose to spend their sabbaticals with us and have kept a commitment thereafter because of their work and experience with training in Namibia. An even more impactful and sustainable approach would be to integrate faculty locally under the umbrella and conditions of the national institution. We reflect that only in this way can global health faculty make a truly sustainable and impactful contribution to remedying the inequalities in health and education that currently exist.
